# High-Throughput Detection of Multiple miRNAs and Methylated DNA by Droplet Digital PCR

**DOI:** 10.3390/jpm11050359

**Published:** 2021-04-29

**Authors:** Ning Li, Pushpa Dhilipkannah, Feng Jiang

**Affiliations:** Department of Pathology, University of Maryland School of Medicine, 10 S. Pine St., Baltimore, MD 21201, USA; lijin6332@gmail.com (N.L.); pdhilipkannah@som.umaryland.edu (P.D.)

**Keywords:** miRNA, methylation, sputum, plasma, lung cancer

## Abstract

Altered miRNA expression and DNA methylation have highly active and diverse roles in carcinogenesis. Simultaneous detection of the molecular aberrations may have a synergistic effect on the diagnosis of malignancies. Herein, we develop a high-throughput assay for detecting multiple miRNAs and DNA methylation using droplet digital PCR (ddPCR) coupled with a 96-microwell plate. The microplate-based ddPCR could absolutely and reproducibly quantify 15 miRNAs and 14 DNA methylation sites with a high sensitivity (one copy/µL and 0.1%, respectively). Analyzing sputum and plasma of 40 lung cancer patients and 36 cancer-free smokers by this approach identified an integrated biomarker panel consisting of two sputum miRNAs (miRs-31-5p and 210-3p), one sputum DNA methylation (RASSF1A), and two plasma miRNAs (miR-21-5p and 126) for the diagnosis of lung cancer with higher sensitivity and specificity compared with a single type of biomarker. The diagnostic value of the integrated biomarker panel for the early detection of lung cancer was confirmed in a different cohort of 36 lung cancer patients and 39 cancer-free smokers. The high-throughput assay for quantification of multiple molecular aberrations across sputum and plasma could improve the early detection of lung cancer.

## 1. Introduction

Lung cancer is the number one cancer killer in the United States and worldwide. Non-small cell lung cancer (NSCLC) accounts for approximately 85% of all lung cancer cases. Tobacco smoking is the major cause of the disease. An NCI-National Lung Screening Trial (NLST) showed that the early detection of lung cancer by low-dose computed tomography (LDCT) could reduce the mortality [1]. However, 25% of smokers screened by LDCT have indeterminate pulmonary nodules (PNs), of which 95% are determined to be false positives [1]. Given the high false positive rate of LDCT, there are large number of referrals for invasive and expensive biopsies that carry their own morbidity. Therefore, the development of noninvasive biomarkers that can accurately diagnose early stage lung cancer remains clinically important [2].

Sputum is a noninvasively and easily accessible body fluid that contains exfoliated bronchial epithelial cells [3]. The lung tumor-related molecular changes in sputum may provide noninvasive biomarkers for the diagnosis of NSCLC [4,5,6,7,8,9,10,11,12,13,14,15,16,17]. MicroRNAs (miRNAs) are the most abundant class of non-coding RNAs, and their dysregulation plays a crucial role in tumorigenesis [18]. We have identified 15 miRNAs that displayed dysregulation in NSCLC [11,15,17]. We have demonstrated that the miRNAs are measurable in sputum by using quantitative PCR (qPCR), and thus provide potential biomarkers for NSCLC [11,13,14,15,17]. Furthermore, plasma biomarkers are based on circulating molecules directly released from lung tumors and floating cancer cells and have been developed for early lung cancer detection [19]. We have shown that miRNAs in plasma provide circulating biomarkers, and further identified a panel of plasma miRNA biomarkers for NSCLC [19,20,21]. In addition, CpG dinucleotides are in the promoter region of tumor suppressor genes (TSGs) [22]. Aberrant promoter methylation can affect the genes involved in cell-cycle control, DNA repair, cell adhesion, signal transduction, apoptosis, and cell differentiation [23]. Epigenetic changes are early events in the carcinogenesis of NSCLC, and thus show great promise as biomarkers for lung cancer [22,24,25]. For instance, we have shown that methylation analysis of *RASSF1A*, *3OST2*, and *PRDM14* could produce 83% sensitivity and 76% specificity for the early detection of NSCLC [24,25,26].

However, two major obstacles exist in the translation of sputum and plasma biomarkers into the clinical settings for the noninvasive diagnosis of lung cancer. First, NSCLC is a heterogeneous disease and develops from complex molecular aberrations through various mechanisms. Dysregulations of miRNAs and DNA methylation contribute to lung tumorigenesis via diverse pathways. Analysis of a single type of molecular biomarkers in either sputum or plasma may not provide sufficient diagnostic significance. Indeed, our individual panels of sputum miRNA, plasma miRNA, or sputum DNA methylation biomarkers used alone had moderate sensitivity (76–86%) and specificity (83–89%) for detection of lung cancer [14,20,25]. Secondly, although conventional qPCR is commonly used for the detection of miRNAs and DNA methylation [8], it is an indirect and labor-consuming approach. We have shown that droplet digital PCR (ddPCR) can absolutely and quantitatively measure nucleic acids and simplifies experimentation and data comparability without requiring internal control genes and calculating standard curves [8,24,25,27,28,29]. Furthermore, ddPCR had a higher sensitivity compared with conventional PCR for the detection of nucleic acids [8,24,25,27,28,29]. However, similarly to conventional qPCR, ddPCR for the detection of multiple types of molecular targets in parallel is time-consuming and labor-intensive and has poor reproducibility. To overcome the two obsoletes, herein we develop a high-throughput assay for the simultaneous and reproducible detection of multiple miRNA and DNA methylation biomarkers in a 96-well plate by using ddPCR. 

## 2. Materials and Methods

### 2.1. Synthetic miRNAs and cDNA

Synthetic single-stranded RNA oligonucleotides corresponding to human mature miRNAs were purchased from Integrated DNA Technologies (Integrated DNA Technologies, San Diego, CA, USA). The varying dilutions of each oligonucleotide were made in H_2_O. cDNA of the input RNA samples was created by using a T100 thermal cycler (Applied Biosystems, Foster City, CA, USA) with the Universal cDNA synthesis kit II (Exiqon, Woburn, MA, USA) as previously described [4,5,6,8,12,13,14,15,17,19,20,21,24,30,31,32,33,34,35,36,37,38]. Briefly, reaction buffer, enzyme mix, RNA, and nuclease-free water were mixed in 20 µL reaction, and incubated for 60 min at 42 °C. The reaction was stopped by inactivating the reverse transcriptase for 5 min at 95 °C. The generated cDNA was diluted in nuclease-free water by 1:50, before being subjected to the next step.

### 2.2. Methylated and Unmethylated DNA and Serially Diluted DNA Samples

We purchased 100% methylated and 100% unmethylated control human DNA samples (Zymo Research, Irvine, CA, USA). To determine the limit of detection (LOD) of an assay, we diluted methylated DNA into unmethylated DNA in different ratios (0, 0.1, 0.5, 1, 2.5, 5, 10, 25, 50, and 100% methylated DNA).

### 2.3. Study Population

The study protocol was approved by the Institutional Review Board of the University of Maryland Medical Center (IRB HP-00040666). Participants between the ages of 55 and 79 at the point of their referral for suspected lung cancer were sought. A total of 76 NSCLC patients and 75 cancer-free smokers were recruited. Among the cancer patients, 26 patients had stage I NSCLC, 25 with stage II, and 25 with stage III–IV. There were no significant differences in age, gender and smoking status between the NSCLC patients and cancer-free smokers. The cases and controls were grouped into two cohorts: a development cohort and a validation cohort. The development cohort consisted of 40 lung cancer patients and 36 cancer-free smokers, while the validation cohort comprised 36 lung cancer patients and 39 cancer-free smokers. The demographic and clinical variables of the two cohorts are shown in Table 1 and Table 2.

### 2.4. Sputum Collection and Preparation

Sputum was collected from the participants before they received any treatment, as described in our previous studies [4,5,6,7,8,9,10,11,12,13,14,15,16,17,37,38,39,40]. Within 2 h of collection, the samples were processed on ice in 4 volumes of 0.1% dithiothreitol (Sigma-Aldrich, St. Louis, MO, USA) followed by 4 volumes of phosphate-buffered saline (PBS) (Sigma-Aldrich). The cell suspension was filtered through 45 µm nylon gauzes (BNSH Thompson, Scarborough, ON, Canada). Absolute cell numbers and cell viability were quantitated by using a hemacytometer with trypan blue. Two cytocentrifuge slides were prepared from aliquots of cell suspension by using a cytospin machine (Shandon, Pittsburgh, PA, USA) and then stained with the Papanicolaou staining technique [3]. A sputum sample was considered adequate if lung macrophages or Curschmann spirals were present on the slides [3,22]. The cell pellets were collected and stored at −80 °C until use.

### 2.5. Plasma Collection and Preparation

Peripheral blood (10 mL) was drawn from the subjects using standardized phlebotomy procedures in BD K2EDTA Tubes (BD, Franklin Lakes, NJ, USA), as described in our previous studies [20,28,36]. The blood specimens were processed within 2 h of collection by centrifugation at 1300× *g* at for 10 min 4 °C. Plasma was transferred to a fresh tube and stored at −80 °C until use.

### 2.6. Isolation of RNA from Sputum or Plasma and Generation of cDNA

Trizol LS reagent (Invitrogen, Carlsbad, CA, USA) and RNeasy Mini Kit (Qiagen, Hilden, Germany) were used to extract RNA from sputum or plasma as previously described [11,13,14,15,17,20]. Eluted RNA (20 ng) was pretreated with one unit of DNase (Invitrogen) and 0.38 μL of RNase inhibitor (Invitrogen). The Universal cDNA synthesis kit II (Exiqon, Woburn, MA) was used to convert RNA to cDNA by using a developed protocol [4,5,6,7,8,12,13,14,15,17,19,20,21,24,30,31,32,33,34,35,36,37,38]. The generated cDNA samples were diluted 1:50 in H_2_O before being used in PCR analyses.

### 2.7. DNA Isolation from Sputum and Bisulfite Conversion

DNA was extracted from sputum using the Qiagen DNeasy kit (Qiagen, Germantown, MD, USA) as previously described [24,25]. We eluted 1 µg DNA with 50 µL of elution buffer (10 mmol/L Tris-Cl, pH 8.5) (Sigma-Aldrich Corporation). Bisulfite conversion was carried out on DNA by using the Zymo EZ DNA Methylation Kit (Zymo Research) in 50 µL elution buffer according to the manufacturer’s protocol.

### 2.8. Pre-Spotted 96-Well Plate with Primer Sets for Multiple miRNA and Methylated Alleles

We previously identified 15 miRNAs whose aberrant expressions were associated with lung cancer [15,17,33,40]. The miRNAs miRs-205-5p, 708-5p, 375, 200b-3p, 182-5p, 155-5p, 372-3p, 143-3p, 486-5p, 126-5p, 31-3p, 21-5p, 210-3p, 135-5p, and 30a-3p were included in this study.

Fourteen genes were also selected for DNA methylation analysis, because the genes were previously reported as potential sputum methylation biomarkers for lung cancer [24,25,26,41,42,43,44,45,46,47,48,49,50,51,52,53,54,55,56,57,58,59,60,61,62]. The 14 genes were 3OST2, APC, DAPK, FHIT, GATA, HOXA9, MAGE, p16, PAX5, DLC1, PRDM14, RASSF1A, SOX17, and TAC1. Sixteen sets of forward and reverse PCR primers for 15 miRNAs and U6 were designed using miRCURY LNA Custom PCR Panel (Qiagen, Germantown, MD) and purchased from Qiagen. Fifteen 15 sets of forward and reverse PCR primers for 14 methylated DNA targets and unmethylated DNA were designed as described in previous studies [4,5,6,7,8,12,13,14,15,17,19,20,21,24,25,26,30,31,32,33,34,35,36,37,41,42,43,44,45,46,47,48,49,50,51,52,53,54,55,56,57,58,59,60,61,62] (Supplementary Table S1).

**Figure 1:**
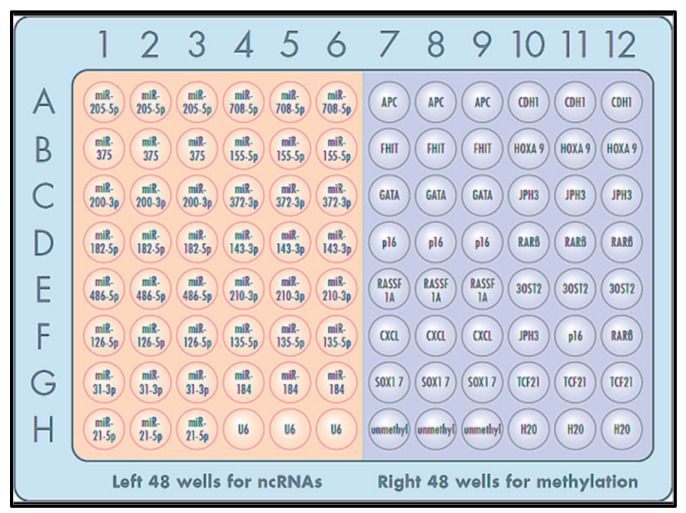
The 96-well microplate was pre-spotted with PCR primer sets for miRNAs (**left** 48 wells) and DNA methyaltion (**right** 48 wells). Each target was run in triplicate.

One microliter of each pair of forward and reverse primers was pre-spotted in a 96-well plate in triplicate, as shown in Figure 1. Primer sets for miRNA and methylated DNA targets were pre-spotted in the left 48 wells and right 48 wells of a 96-well microplate, respectively (Figure 1). The remaining three wells were loaded with 1 µL H_2_O.

### 2.9. Simultaneous Detection and Quantification of miRNAs and DNA Methylation

**Figure 2:**
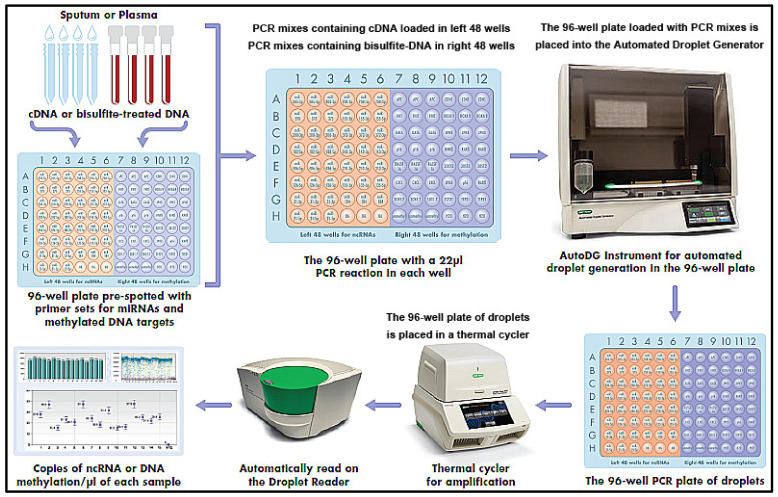
The workflow of the microplate-based ddPCR system.

The workflow of the detection system is shown in Figure 2.

A 22 µL volume of PCR mix containing either diluted cDNA or bisulfite-treated DNA was prepared from sputum and plasma. PCR primer sets for miRNAs and methylated DNA were pre-spotted in the left 48 wells and right 48 wells of the plate; therefore, the PCR mixes containing cDNA for miRNAs were loaded in left 48 wells, while the PCR mixes containing bisulfite-treated DNA were in right 48 wells of the plate, respectively, to ensure that miRNAs and DNA methylations were specifically targeted. A bottle of Automated Droplet Generation Oil (Bio-Rad, Hercules, CA, USA) was loaded into an Automated Droplet Generator (Bio-Rad), along with DR32 Cartridges (Bio-Rad) and pipette tips for the Automated Instrument. The plate was placed into the Automated Droplet Generator. In the system, the 22 µL of the PCR reaction was automatically transferred to each well of the cartridge using a multichannel pipette and then filled with 70 µL DG oil for EvaGreen^®^ (Biotium, Inc., Fremont, CA, USA). The gasket was hooked over the cartridge holder, which was robotically inserted into the QX200™ Droplet Generator (Bio-Rad), to generate droplets. After the droplet generation, the 96-well PCR plate of droplets was placed in a T100 thermal cycler (Applied Biosystems). Thermal cycling conditions for all EvaGreen assays included an activation step (5 min at 95 °C) followed by 40 cycles of a two-step thermal profile, comprising a denaturation step (30 s at 95 °C) and a combined annealing extension step (60 s at 60 °C). A dye-stabilization step was then performed that consisted of 4 °C for 5 min, then 95 °C for 5 min, and finally, was maintained at 4 °C. After completion, the plate was transferred to a droplet reader (Bio-Rad). By using the software (Bio-Rad) with Poisson distribution analysis, we computed the copy number of miRNAs or methylated DNA/µL from the fraction of positive reactions, as previously described [8,24,25,27,28,29,33,40,63]. The numbers of positive and total generated droplets were calculated. The well contained more than 10,000 droplets, which suggested that it could be successfully “read” by passing the droplets through a fluorescence detector.

### 2.10. Statistical Analysis

We used statistical system software version 6.12 (SAS Institute, Cary, NC, USA) and GraphPad Prism version 7 (GraphPad Software, La Jolla, CA, USA) for data analysis. The results were graphed and plotted by GraphPad Prism version 7. A Mann–Whitney U test was used to determine whether the result of the targeted genes were significantly different between lung cancer patients and healthy controls. Furthermore, Pearson’s correlation coefficient test was used to determine the associations of the genes with clinicopathologic and demographic characteristics of the participants, as well as agreements between the results from different tests and on different days. Spearman’s correlation test was carried out to analyze the correlation between levels of the genes. Logistic regression was used to generate prediction models. To evaluate the diagnostic significance of potential biomarkers, we used receiver–operator characteristic (ROC) curve analysis and computed the area under the ROC (AUC) value by numerical integration of the ROC curve.

## 3. Results

### 3.1. The Microplate-Based ddPCR Has a High Analytic Performance for Quantifying Multiple miRNAs and DNA Methylations

**Figure 3:**
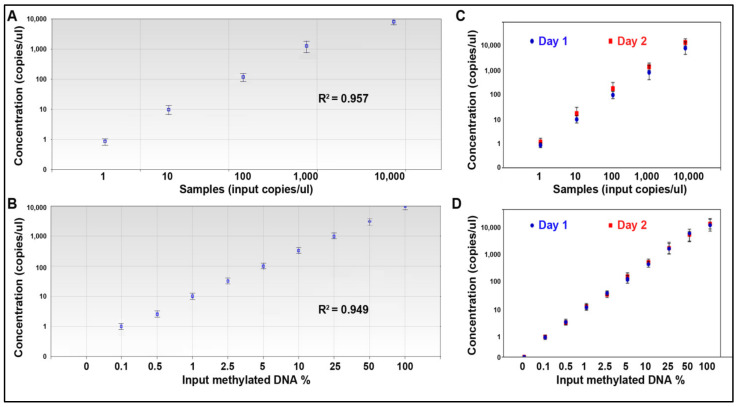
The analytic performance of microplate-based ddPCR for the quantification of miRNAs and DNA methylation. (**A**) Linearity of miR-31-5p concentration measured by microplate-based ddPCR in a dilution series of known input amounts of synthetic oligonucleotide corresponding to miR-31-5p. Microplate-based ddPCR had a dynamic range of 5 orders of magnitude from 1 to 10,000 copies per µL. (**B**) Linearity of RASSF1A methylation measured by microplate-based ddPCR in serially diluted methylated DNA samples suggests that the test has a dynamic range of 9 orders of magnitude from 0.1 to 100%. (**C**) The analysis of the miRNAs by microplate-based ddPCR on different days had a high agreement between the results (Pearson r = 0.91). (**D**) The analysis of DNA methylation by microplate-based ddPCR on different days had a high agreement between the results (Pearson r = 0.93). All the 15 miRNAs and 14 methylation were tested by microplate-based ddPCR, producing the same analytic performance. The figure only shows the results of miRs-31-5p and RASSF1A methylation.

The serially diluted cDNA and bisulfite-treated DNA samples were run for quantification of the 15 miRNAs and 14 DNA methylation events. Each well of the samples generated at least 10,000 droplets. There was excellent linearity between the input miRNA and values determined by ddPCR (R^2^ = 0.957) across five orders of magnitude for all the miRNAs (Figure 3A). ddPCR could detect the miRNAs levels down to the limit of detection (LOD) (1 copies/µL). There was also excellent linearity between the methylated DNA input and values measured by ddPCR (R^2^ = 0.949). Furthermore, the assay detected methylated genes at a concentration of 0.1% (LOD = 0.1%) (Figure 3B).

To determine reproducibility of the approach for quantification of the molecular events, each of the diluted samples were aliquoted and tested on different days. There was a high agreement between the results of miRNAs detected on different days (Pearson r = 0.91) (Figure 3C). There was also a high agreement between the results of DNA methylation detected on different days (Pearson r = 0.93) (Figure 3D). In addition, the whole process of the test took only 4 h. Therefore, the microplate-based ddPCR has high analytic sensitivity and reproducibility for simultaneously quantifying multiple miRNAs and DNA methylations.

### 3.2. Diagnostic Performance of the Microplate-Based ddPCR for Lung Cancer

**Figure 4:**
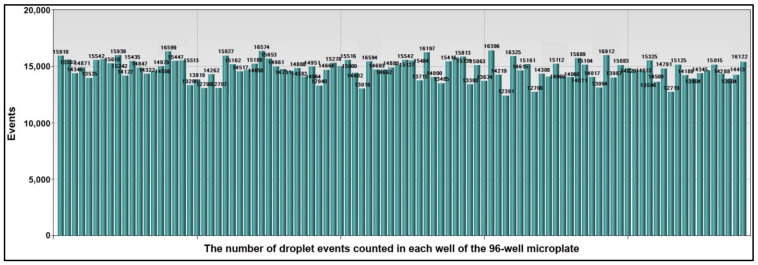
The number of droplet events counted in each well of the 96-well microplate. A total of 15 miRNAs in the plasma sample and 14 methylated DNA targets in the sputum of one lung cancer patient were quantified by microplate-based ddPCR. Each column represented one well of the 96-well microplate. Each well contained at least 10,000 droplets.

We used microplate-based ddPCR to quantify the 15 miRNAs in both sputum and plasma and 14 DNA methylations in the sputum of the development cohort of 40 NSCLC patients and 36 cancer-free subjects (Table 1). All the miRNAs and DNA methylation events generated more than 10,000 droplets per well (Figure 4). Therefore, sputum could be successfully “read” by passing the droplets through a fluorescence detector and the molecular targets could be reliably detected in the clinical specimens by this approach.

**Figure 5:**
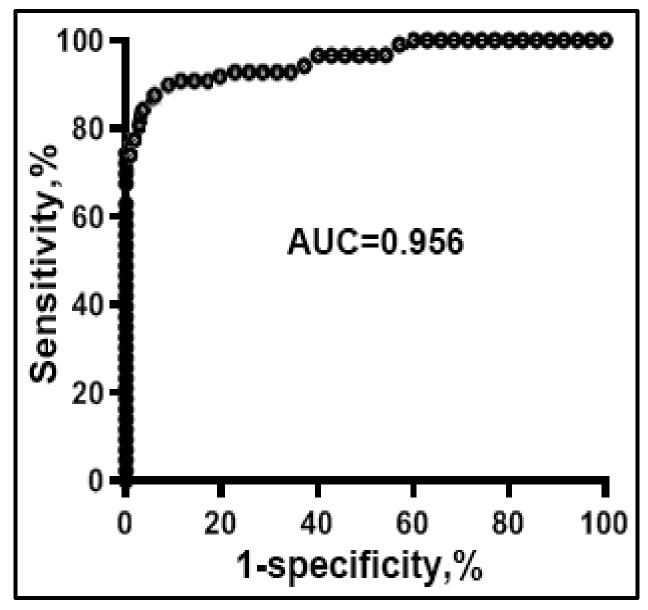
Diagnostic performance of combined use of two sputum miRNA (miRs-31-5p and 210-3p), one sputum DNA methylation (RASSF1A), and two plasma miRNA (miR-21-5p and 126) biomarkers. ROC curve of the panel of biomarkers shows an AUC of 0.95 for differentiating NSCLC patients from the cancer-free subjects in terms of sensitivity and specificity.

The 15 sputum miRNAs, 14 sputum DNA methylation events, and 15 plasma miRNAs displayed statistically different copy numbers in the sputum of lung cancer patients vs. cancer-free smokers (Supplementary Table S2) (all *p* < 0.05). Particularly, 10 miRNAs or 5 miRNAs had a significantly higher or lower copy number in the sputum of lung cancer patients compared with cancer-free controls, respectively (Supplementary Table S2) (all *p* < 0.05). All miRNAs had a significantly higher copy number in the plasma of lung cancer patients compared with cancer-free controls (all *p* < 0.05). Fourteen displayed a higher methylation level in the sputum of lung cancer patients vs. controls (all *p* < 0.05). The individual miRNAs in the sputum, plasma and DNA methylation in sputum showed AUC values of 0.61–0.85 for differentiating lung cancer cases from control subjects (Supplementary Table S2). We used LASSO and AUCs to determine performance of different patterns of combining the miRNA and DNA methylation biomarkers. Two sputum miRNAs (miRs-31-5p and 210-3p), one sputum DNA methylation (RASSF1A), and two plasma miRNAs (miR-21-5p and 126) were identified and optimized as a biomarker panel, producing 0.96 AUC (Figure 5), which was statistically higher than that of any single molecular changes (*p* < 0.05).

As a result, the use of the panel of integrated biomarkers generated 92.50% sensitivity and 91.67% specificity for the detection of lung cancer. Spearman’s correlation test indicated that the estimated correlations among levels of the two miRNAs in sputum and two miRNAs in plasma and one DNA methylation site in sputum were low (all *p* > 0.05). The copy numbers of the two sputum miRNAs and one sputum DNA methylation were associated with the smoking history of participants (Pearson’s correlation coefficient test, all *p* < 0.05) and SCC of the lungs (*p* < 0.05). However, overall, the panel of the biomarkers did not exhibit special association with the stage or histology of lung cancer, or age, gender, and ethnicity of the participants (Pearson’s correlation coefficient test, all *p* > 0.05).

We then evaluated the diagnostic performance of the integrated biomarker panel in specimens of the validation cohort of 36 NSCLC patients and 39 healthy controls (Table 2). The panel of integrated biomarkers could diagnose NSCLC with 91.67% sensitivity and 92.31% specificity. Furthermore, no statistically significant difference was found in the sensitivity and specificity of the biomarkers for stages and histological types of NSCLC (all *p* > 0.05). Moreover, there was no association of expressions of the biomarkers with the age, gender, or smoking status of the lung cancer patients and normal individuals (all *p* > 0.05). Taken together, the results confirmed the diagnostic significance of the panel of biomarkers for the early detection of lung cancer.

## 4. Discussion

Interaction between miRNAs and DNA methylation and their diagnostic values have been extensively investigated in a variety of cancer types [64,65,66,67,68]. For instance, Konno et al. suggested that certain miRNAs harbor methyl marks that could modify their stability and target recognition [66]. Methylation of these miRNAs was significantly increased in gastrointestinal cancer tissues as compared to paired normal tissues. Their findings might provide diagnostic strategies of using miRNAs for early stage cancer [66]. Using bioinformatic approach and selection strategies, Tang et al. identified tissue-specific CpG sites and deduced the classifiers that could predict the origin of tumors [65]. Padrão et al. found that promoter methylation levels of miR-129-2 and miR-663a could be used as biomarkers for the diagnosis of urothelial carcinoma [68]. Moreover, the study from Nakaoka et al. suggested that aberrant DNA methylation of tumor suppressor genes and miRNAs could be used for the diagnosis and treatment of cholangiocarcinoma [67]. We previously investigated correlations between DNA methylation and miRNA expression and function in lung cancer, and found that interaction between miRNAs and DNA methylation could have synergistic effects on lung tumorigenesis [64]. Furthermore, we found that combined analysis of different molecular events (e.g., miRNAs and DNA methylation) had higher diagnostic significance than a single molecular type [24]. Although these previous studies documented the power of analyzing aberrant DNA methylation of miRNAs in the diagnosis of cancers, simultaneous detection of the two important molecular events across different types of specimens still remains a technical challenge.

To overcome this challenge, in this present study, we used the QX200 AutoDG ddPCR System (Life Science, Hercules, CA) coupled with a 96-microwell plate to quantify multiple types of molecular targets in two different sample types, sputum and plasma. The system can simplify the ddPCR workflow, make experiments both scalable and practical, and minimize hands-on time. It also has a high-efficiency particulate air-filtered enclosure that could reduce the possibility of contamination and allow the system to be placed in any lab environment. In addition, the system eliminates user-to-user variability, allowing full exploitation of the precision of ddPCR technology by providing more consistent droplet counts across users and plates. We found that the microplate-based ddPCR assay has high reproducibility for the quantification of both miRNAs and DNA methylation. The use of microplate-based ddPCR could simplify the complexity of combined analysis of different specimen types, while maximizing their potentials for lung cancer diagnosis. Results of different classes of molecular changes generated from different sample types could be directly and automatically read and presented in the same format as copy numbers of miRNAs or methylated DNA/µL in each well (or sample). The whole process of the test takes only 4 h. Furthermore, the arrangement of numbers of PCR primer sets is adjustable, depending on the molecular targets to be detected. For instance, if each target is tested in duplicate, a composition of either (a) 48 miRNA, (b) 48 methylation, or (c) combing 24 miRNAs and 24 methylations can be simultaneously qualified in sputum and plasma. In addition, the possibility of extending this approach to simultaneously assess other important molecular events, such as DNA mutations and long non-coding RNAs in a lager microplate (e.g., 486-well plate), would support and improve disease biomarker discovery and validation. Therefore, this high-throughput assay for the simultaneous and sensitive detection of multiple miRNAs and methylated DNA across different sputum and plasma might represent a new and robust approach for the diagnosis of lung cancer.

Establishing the optimal annealing temperature could be a challenge among individual primers sets when multiple targets are amplified at the same time in a single plate. To overcome this obstacle, we used the miRNA LNA PCR primers that are optimized to work with EvaGreen^®^ supermix (Biotium, Inc. Fremont). We also used EvaGreen-based methylation-specific PCR primers that work well in the same conditions as the miRNA LNA PCR primer sets. Using the uniform thermal cycling condition and the same amount of primer, we could quantify copy numbers of miRNAs and DNA methylation at the same time, producing high specificity, sensitivity, and reproducibility.

By using this new approach, we further developed a panel of integrated biomarkers, consisting of sputum miRNAs (miRs-31-5p and 210-3p), one sputum DNA methylation (RASSF1A), and two plasma miRNAs (miR-21-5p and 126). Combined use of the multiple classes of molecular events yielded a higher diagnostic performance compared with a single type of the molecular biomarkers. Furthermore, the correlations among the changes of the miRNA and DNA methylation biomarkers were very low, supporting the assertion that the diagnostic vales of the two classes of molecular alterations tested in different sample types could be complementary to each other. In addition, sputum biomarkers were developed from exfoliated bronchial epithelia of large airways or main bronchi where SCC commonly exists, and thus are more sensitive for the diagnosis of SCC. In contrast, plasma biomarkers are based on circulating molecules directly released from lung tumors and floating cancer cells, and thus are more sensitive for AC. Our present study shows that combining sputum and plasma could be used to diagnose NSCLC, regardless of the histologic types and stages. Furthermore, the observations that the biomarkers are independent of stages and histological types of lung cancer were validated in a different cohort of cases and controls. Therefore, the findings confirmed our hypothesis that combined analysis of the multiple classes and targets of molecular changes in sputum and plasma might provide synergistic value for the early detection of lung cancer, particularly stage I NSCLC.

This study may suffer from some limitations. Firstly, the existing samples were retrospectively collected, which might have produced selection bias and overfitting for the development of biomarkers. We will perform a large and prospective study to validate the diagnostic performance of the biomarkers. Secondly, the development of the biomarkers for specifically identifying NSCLC in an LDCT positive screening setting will reduce lung cancer mortality by sparing smokers with benign PNs from invasive and expense multiple follow-up examinations, facilitating effective treatments to be instantly initiated for NSCLC [1]. However, the participants in this current study might not be representative of the smokers in LDCT screening settings for lung cancer. We will perform a prospective trial to determine if the sputum biomarkers could be used as an effective screening test for specifically identifying NSCLC in an LDCT screening positive setting.

## 5. Conclusions

We have developed a high-throughput platform for simultaneously and efficiently quantifying multiple molecular changes in a 96-well plate by using ddPCR. We further demonstrated that the integration of DNA methylation and miRNA biomarkers across sputum and plasma could provide a synergistic effect for the diagnosis of early stage lung cancer. Nonetheless, a large, multi-center clinical project to further validate the approach is required before the platform and biomarkers could be adopted in routine clinical settings.

## Figures and Tables

**Table 1:** A development cohort of NSCLC patients and cancer-free smokers from whom sputum and plasma were collected.

	NSCLC Cases (*n* = 40)	Controls (*n* = 36)	*p*-Value
Age	65.29 (SD 10.01)	63.23 (SD 9.78)	0.33
Sex			0.65
Female	14	12	
Male	26	24	
Race			0.49
African Americans	12	10	
White American	28	26	
Smoking pack-years (median)	34.16	29.46	0.22
Stage			
Stage I	13		
Stage II	13		
Stage III–VI	14		
Histological type			
Adenocarcinoma	22		
Squamous cell carcinoma	18		

Abbreviations: NSCLC, non-small cell lung cancer.

**Table 2:** A validation cohort of NSCLC patients and cancer-free smokers from whom sputum and plasma were collected.

	NSCLC Cases (*n* = 36)	Controls (*n* = 39)	*p*-Value
Age	66.37 (SD 10.23)	62.25 (SD 9.16)	0.32
Sex			0.54
Female	12	14	
Male	24	25	
Race			0.46
African Americans	10	13	
White American	26	26	
Smoking pack-years (median)	35.11	31.47	0.29
Stage			
Stage I	13		
Stage II	12		
Stage III-VI	11		
Histological type			
Adenocarcinoma	20		
Squamous cell carcinoma	16		

Abbreviations: NSCLC, non-small cell lung cancer.

## Supplementary Materials

The following are available online at https://www.mdpi.com/article/10.3390/jpm11050359/s1. Table S1: Primers for ddPCR analysis of 14 methylation sites. Table S2: 15 sputum miRNAs, 14 sputum DNA methylation, and 15 plasma miRNAs display a difference level in lung cancer patients.
