# Supplementary material for: High-Throughput Detection of Multiple miRNAs and Methylated DNA by Droplet Digital PCR
Source: J Pers Med. 2021 Apr 29;11(5):359. doi: 10.3390/jpm11050359 (PMC8146424; doi:10.3390/jpm11050359)
Supplement: Supplementary file 1 [file jpm-11-00359-s001.zip › jpm-1156454-supplementary.pdf]

Supplementary table 1. Primers for ddPCR analysis of 14 methylation sites

Genes	Primer sequence (5′-3′) forward	Primer sequence (5′-3′) reverse
3OST2	CGGTTGTTCGGAGTTTTATC	GTAACGCTACCACGACCACG
APC	TATTGCGGAGTGCGGGTC	TATTGCGGAGTGCGGGTC
DAPK	GGATAGTCGGATCGAGTTAACGTC	CCCTCCCAAACGCCGA
FAM19A4	CGGGCGGTTCGGTTAATT	AAAACGACGCGCAACTAAC
FHIT	TTTTCGTTTTTGTTTTTAGATAAGC	AAAAATATACCCACTAAATAACCGC
GATA	GTATAGTTTCGTAGTTTGCGTTTAGC	AACTCGCGACTCGAATCCCCG
H-cadherin	TAATTTTAGGTTAGAGGGTTATTGT	CACAACCAATCAACAACACA
HOXA9	GGTTAATGGGGGCGCGGGCGTC	AACGCCTAACCCGCCCGACCCG
JPH3	AGACGTTGGTTAGGTTTCGC	CGCGACCCCGAAACGCG
MAGE	TTTCGACGTTCGTAGGTTTTCGC	GCACTCTTCCGAAAACGAAACG
p16	TTATTAGAGGGTGGGGCGGATCGC	GACCCCGAACCGCGACCGTAA
PAX5	TTCGCGTATAGCGTAGAGGGTCG	AAACGTAACGAACCCGACCCG
DLC1	TTTAAAGATCGAAACGAGGGAGCG	CCCAACGAAAAAACCCGACTAACG
PRDM14	GGTAGTAAATAAATTTAGTAGTTGCGT	CCCTAAAACTAAAATCCTACGTA
RARβ	GTTAAAGGGGGGATTAGAAT	CACGCATACGCGAAACAACTG
RASSF1A	ACGCGTTGCGTATCGCGCG	CCGCGACGACTACGCTACC
SOX17	GGGTTTTTAAAATTTTCGCGTCGC	CTACGAAACCGAACGAAAATACG
TAC1	GGCGGTTAATTAAATATTGAGCAGAAAGTCGC	AAATCCGAACGCGCTCTTTCG

Table 2. 15 sputum miRNAs, 14 sputum DNA methylation, and 15 plasma miRNAs display a difference level in lung cancer patients vs. controls

	Sample type	Level in NSCLC patients, mean (SEM)	Level in Controls, mean (SEM)	P-value	AUC	Sensitivity (%)	Specificity (%)
miR-205-5p	Sputum	82.35 (12.17)	40.77 (28.13)	0.02	0.64	59.87	53.69
miR-708-5p	Sputum	1.09 (0.09)	4.39 (1.21)	0.00	0.66	64.78	62.36
miR-375	Sputum	567.83 (126.25)	56.22 (15.34)	< 0.001	0.67	66.24	62.46
miR-200b-3p	Sputum	821.84 (133.68)	59.76 (12.39)	0.01	0.67	65.26	61.23
MiR-182-5p	Sputum	35.23 (9.76)	5.54 (3.37)	0.00	0.68	64.67	59.83
miR-155-5p	Sputum	20.46(5.79)	6.53 (1.29)	0.00	0.69	62.38	62.28
miR-372-3p	Sputum	121.39 (20.51)	15.47 (4.56)	0.00	0.71	63.66	60.99
miR-143-3p	Sputum	10.42 (2.89)	1.83 (0.64)	0.03	0.73	63.69	61.58
miR-486-5p	Sputum	28.45 (3.48)	232.65 (35.81)	0.00	0.72	74.12	66.37
miR-126-5p	Sputum	6.86 (2.48)	69.88 (20.42)	0.00	0.78	77.65	75.03
miR-31-3p	Sputum	2.98 (0.67)	0.19 (0.16)	< 0.001	0.78	60.38	82.65

miR-21-5p	Sputum	50.35 (5.35)	6.38 (1.09)	< 0.001	0.82	78.16	71.16
miR-210-3p	Sputum	65.23 (5.17)	5.46 (0.53)	< 0.001	0.85	75.28	85.68
miR-135-5p	Sputum	15.38 (5.41)	26.44 (6.51)	0.02	0.80	77.14	71.05
miR-30a-3p	Sputum	27.76 (8.23)	35.46 (9.18)	0.01	0.83	73.29	85.63
miR-205-5p	Plasma	62.42 (8.10)	30.72 (6.45)	0.00	0.72	69.84	73.62
miR-708-5p	Plasma	5.19 (0.13)	1.29 (0.21)	0.02	0.61	62.76	63.36
miR-375	Plasma	65.81 (12.26)	26.25 (5.37)	0.02	0.63	67.21	61.36
miR-200b-3p	Plasma	21.37 (3.18)	9.48 (1.23)	0.03	0.73	69.29	71.25
MiR-182-5p	Plasma	15.26 (4.36)	3.52 (0.94)	0.01	0.64	62.62	61.81
miR-155-5p	Plasma	12.36(4.19)	3.23 (0.79)	0.01	0.62	63.31	62.37
miR-372-3p	Plasma	31.32 (7.54)	6.44 (1.52)	0.00	0.67	62.64	61.23
miR-143-3p	Plasma	6.43 (1.39)	0.73 (0.13)	0.01	0.64	61.65	66.54
miR-486-5p	Plasma	22.12 (5.28)	9.63 (3.82)	0.01	0.72	73.13	68.36
miR-126-5p	Plasma	14.83 (3.45)	5.83 (2.49)	0.01	0.83	77.67	82.12
miR-31-3p	Plasma	1.92 (0.48)	0.08 (0.09)	0.01	0.82	74.33	78.65
miR-21-5p	Plasma	32.27 (6.45)	4.33 (1.52)	0.02	0.84	79.15	83.17
miR-210-3p	Plasma	25.25 (2.16)	4.48 (1.23)	0.00	0.78	76.28	78.62
miR-135-5p	Plasma	12.33 (4.51)	6.43 (2.17)	0.01	0.77	73.35	72.04
miR-30a-3p	Plasma	22.34 (5.25)	35.46 (8.15)	0.00	0.76	70.19	75.68
3OST2	Sputum	12.27 (3.11)	5.73 (2.48)	0.00	0.68	0.55	0.82
APC	Sputum	15.13 (4.15)	3.22 (1.01)	0.02	0.67	0.45	0.86
DAPK	Sputum	14.87 (5.23)	5.26 (2.64)	0.02	0.65	0.46	0.81
FHIT	Sputum	35.32 (9.78)	12.28 (6.43)	0.03	0.75	0.64	0.85
GATA	Sputum	16.22 (5.56)	6.62 (2.34)	0.02	0.64	66.78	57.67
HOXA9	Sputum	16.46(6.59)	10.24 (4.69)	0.02	0.62	78.45	53.23
MAGE	Sputum	11.12 (5.64)	4.57 (1.26)	0.02	0.63	56.75	73.28
p16	Sputum	16.23 (6.30)	5.75 (2.33)	0.02	0.65	59.43	81.85
PAX5	Sputum	12.36 (3.29)	6.66 (3.11)	0.02	0.72	48.76	72.37
DLC1	Sputum	15.85 (4.56)	4.33 (1.79)	0.02	0.73	59.54	58.38
PRDM14	Sputum	10.97 (4.52)	5.78 (2.49)	0.03	0.77	65.59	74.76
RASSF1A	Sputum	5.26 (1.87)	2.34 (0.98)	0.03	0.75	56.87	75.35
SOX17	Sputum	15.15 (5.73)	6.69 (2.26)	0.00	0.78	84.65	69.65
TAC1	Sputum	27.35 (6.79)	10.47 (6.37)	0.02	0.85	88.64	75.55

Abbreviations: SEM, the standard error of the mean; AUC, the area under receiver operating characteristic curve; CI, confidence interval.
